# Positive Association Between Plasma Aldosterone Concentration and White Matter Lesions in Patients With Hypertension

**DOI:** 10.3389/fendo.2021.753074

**Published:** 2021-11-18

**Authors:** Yujuan Yuan, Nanfang Li, Yan Liu, Qing Zhu, Mulalibieke Heizhati, Weiwei Zhang, Xiaoguang Yao, Deilian Zhang, Qin Luo, Menghui Wang, Guijuan Chang, Mei Cao, Keming Zhou, Lei Wang, Junli Hu, Nuerguli Maimaiti

**Affiliations:** ^1^ Hypertension Center of People’s Hospital of Xinjiang Uygur Autonomous Region, Xinjiang Hypertension Institute, National Health Committee Key Laboratory of Hypertension Clinical Research, Key Laboratory of Xinjiang Uygur Autonomous Region “Hypertension Research Laboratory”, Xinjiang Clinical Medical Research Center for Hypertension (Cardio-Cerebrovascular) Diseases, Urumqi, China; ^2^ Xinjiang Medical University, Urumqi, China; ^3^ Radiography Center of People’s Hospital of Xinjiang Uygur Autonomous Region, Urumqi, China

**Keywords:** plasma aldosterone concentration (PAC), white matter lesions (WMLs), hypertension, cerebral small vessel disease, plasma renin activity (PRA)

## Abstract

**Background and Objective:**

White matter lesions (WMLs) are imaging changes in MRI of cerebral small vessel disease associated with vascular risk factors, increasing the risk of dementia, depression, and stroke. Aldosterone (ALD) or activation of mineralocorticoid receptor (MR) causes cerebrovascular injury in a mouse model. We aimed to analyze the relationship between ALD and WMLs in a population with hypertension.

**Methods:**

We conducted a retrospective review of all patients screened for causes of secondary hypertension. We enrolled 547 patients with WMLs and matched these to controls without WMLs at a 1:1 ratio. White matter lesion load was assessed by using a modified Scheltens’ scale.

**Results:**

Among the analytic sample (N = 1,094) with ages ranging from 30 to 64 years, 62.2% were male. We divided plasma ALD concentration (PAC), plasma renin activity (PRA), and ALD–renin ratio (ARR) into the third tertile (Q3), second tertile (Q2), and first tertile (Q1). We also analyzed them simultaneously as continuous variables. Multivariate logistic regression analysis showed that participants in Q3 (>17.26 ng/dl) of PAC (OR 1.59, 95% CI 1.15, 2.19), Q3 (<0.80 ng/dl) of PRA (OR 2.50, 95% CI 1.81, 3.44), and Q3 (>18.59 ng/dl per ng/ml*h) of ARR (OR 2.90, 95% CI 2.10, 4.01) had a significantly higher risk of WMLs than those in Q1 (<12.48) of PAC, Q1 (>2.19) of PRA, and Q1 (<6.96) of ARR. In linear regression analysis, we separately analyzed the correlation between the modified Scheltens’ scale score and log(PAC) (β = 2.36; 95% CI 1.30, 3.41; *p* < 0.001), log(PRA) (β = −1.76; 95% CI −2.09, −1.43; *p* < 0.001), and log(ARR) (β = 1.86; 95% CI 1.55, 2.17; *p* < 0.001), which were all significantly correlated with white matter lesion load, after adjusting for confounding factors. Simple mediation analyses showed that systolic blood pressure (SBP) or diastolic blood pressure (DBP) mediated −3.83% or −2.66% of the association between PAC and white matter lesion load, respectively. In stratified analyses, there was no evidence of subgroup heterogeneity concerning the change in the risk of WMLs (*p* > 0.05 for interaction for all).

**Conclusion:**

Higher PAC, especially in PAC >17.26 ng/dl, increased the risk of WMLs. PAC was positively associated with white matter lesion load independent of SBP or DBP.

## Introduction

White matter lesions (WMLs), also known as white matter hyperintensities (WMHs) or leukoaraiosis refer to hyperintense lesions in the white matter visible on T2-weighted MRI (T2-MRI) and fluid-attenuated inversion recovery (FLAIR) (1, 2). WMLs are an imaging change of cerebral small vessel disease in MRI, referring to pathologies such as chronic cerebral ischemia, hypoperfusion, endothelial dysfunction, blood–brain barrier breakdown, inflammatory response, and genetic factors affecting the small cerebral arteries, arterioles, venules, and capillaries (3). These vascular pathologies eventually lead to tissue damage that can impact brain function, histologically evident as (incomplete or complete) infarcts, demyelination, loss of oligodendrocytes, and/or axonal damage (4).

WMLs increase the risks of dementia (5–7), depression (8), stroke occurrence (9), stroke recurrence (3), and even death (10). Therefore, it is of vital importance to prevent and manage these disabling chronic conditions. Many risk factors, such as age, hypertension, diabetes, hyper-homocysteinemia, anxiety, and hyperlipidemia, are associated with WMLs. Among various risk factors, hypertension is the most important leading to WMLs (11–14). Hypertension is also a higher risk factor for cognitive decline and dementia, including Alzheimer’s disease (AD) (15–18). Recent evidences suggest that inhibition of renin–angiotensin system (RAS) in this system may be beneficial in attenuating cognitive deficits observed in aging, AD, Parkinson’s disease (PD), vascular cognitive impairment, and post-stroke cognitive impairment (19–22). Renin has been identified within neurons and astrocytes (23). In the brain, renin receptor is expressed in cardiovascular regulatory nuclei and is linked to hypertension (24). Low-renin hypertension affects 30% of patients with hypertension (25, 26).

Approximately 5% to 13% of patients with hypertension have accompanying increased secretion of excessive aldosterone (ALD) and activation of the mineralocorticoid receptor (MR) (27, 28). MR medicates the cellular response to ALD. MR is, however, expressed more widely and found in many tissues and cell types, including in the cardiovascular, inflammatory, and central nervous systems (CNS), where the actions of MR extend well beyond the maintenance of sodium and potassium homeostasis (29). Several studies have found that ALD or activation of the MR causes cerebrovascular injury in mice (30–32), and these injuries are prevented by spironolactone treatment in endothelial cell MR-deficient mice (32). Verpillat et al. also found an association between ALD synthase (*CYP11B2*), a key enzyme gene for ALD synthesis, and WMLs seen on cerebral MRI independent of hypertension (33). The association between ALD and WMLs has been widely shown in animal models but is still unclear in humans.

Therefore, it is necessary to analyze the association of ALD and WMLs in order to understand the damage of ALD to the cerebral small vessel. The current study investigated the relationship between ALD and WMLs and analyzed the association between plasma ALD concentration (PAC), plasma renin activity (PRA), and white matter lesion load in patients with hypertension.

## Materials and Methods

### Study Population

**Figure 1:**
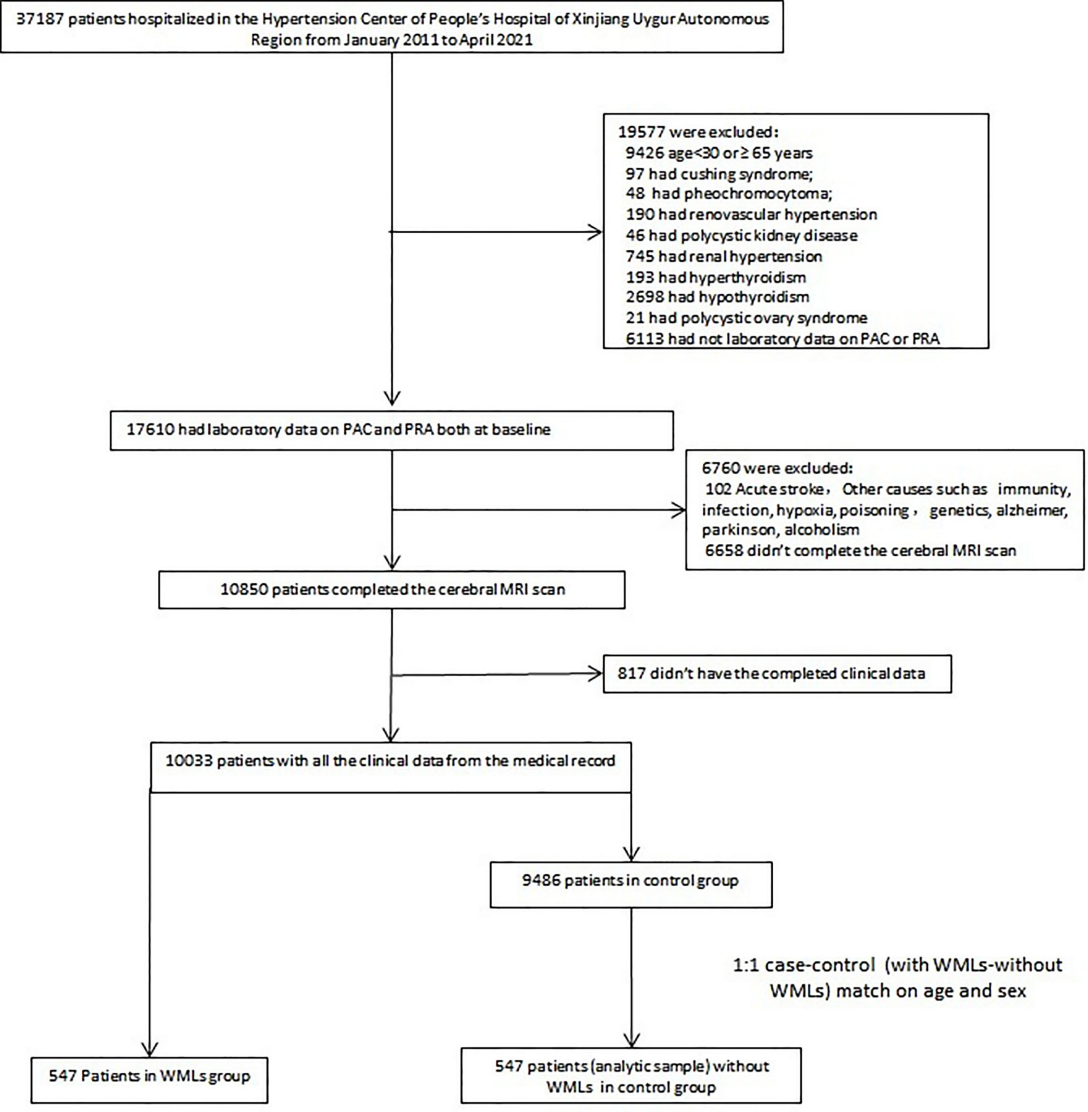
Flowchart visualizing the selection process of the patients. PAC, plasma renin activity; PRA, plasma aldosterone concentration; WMLs, white matter lesions.

We conducted a retrospective review of all patients screened for causes of secondary hypertension in the Hypertension Center of the People’s Hospital of Xinjiang Uygur Autonomous Region between January 1, 2011, and April 1, 2021. We employed an internal database search engine, YiDu Cloud, to identify patients of interest based on the following entry criteria: admission to Hypertension Center; age from 30 to 65 years; cerebral MRI scan results; and laboratory data on PAC and PRA both at baseline. A total of 547 patients with WMLs and 9,486 patients without WMLs were included. Among the analytic sample, patients with WMLs (n = 547) were matched with patients without WMLs (1:1 ratio) by age and sex (**Figure 1**). All patients were requested to discontinue angiotensin-converting enzyme inhibitors, angiotensin receptor blockers, dihydropyridine-calcium antagonists, and β-receptor blockers for at least 4 weeks or diuretics and mineralocorticoid antagonists for at least 6 weeks or were not taking any antihypertensive medications at least 2 weeks before the PAC and PRA measurements. Premenopausal women were not taking any oral contraceptives, and the postmenopausal women were not taking any hormone replacement therapy. When necessary, antihypertensive agents were replaced with slow-release verapamil or α1 epinephrine antagonists (doxazosin or terazosin) or a combination of the two to minimize interference with the measurement of PAC and PRA.

Thus, all included study participants received a full medical examination, consisting of complete medical record, standardized clinical examination, a cerebral MRI scan, and a blood test. The study was approved by the Ethics Committee of the People’s Hospital of Xinjiang Uygur Autonomous Region.

The inclusion criteria were as follows:

1. WML group: patients were examined by MRI, and the results showed hyperintense lesions in the white matter visible on T2-MRI and FLAIR.
2. Control group: hyperintense lesions in the visible white matter were not seen on T2-MRI or FLAIR.

The exclusion criteria were as follows:

1) Cushing’s syndrome; 2) pheochromocytoma; 3) renovascular hypertension; 4) renal hypertension; 5) polycystic kidney disease; 6) hyperthyroidism; 7) hypothyroidism; 8) polycystic ovary syndrome; 9) acute stroke; 10) other causes such as immunity, infection, hypoxia, poisoning, and genetic conditions, such as Sneddon syndrome, Huntington, neurofibromatosis, leukodystrophies, multiple sclerosis, lupus, and sickle cell disease; and 11) any diseases affecting cognitive function, including AD, PD, and alcoholism.

### Clinical Data Collection

We collected all the clinical data from the medical records of study participants, including age, sex, body weight and height, smoking or alcohol consumption status, systolic blood pressure (SBP), diastolic blood pressure (DBP) on admission, duration of hypertension, medical diagnoses, history of diabetes mellitus, coronary artery disease (CAD), creatinine (Cr), hemoglobin A1c (HbA1c), fasting blood glucose (FBG), PRA, PAC, triglyceride (TG), total cholesterol (TC), low-density lipoprotein-cholesterol (LDL-C), serum potassium and homocysteine (Hcy), adrenocorticotropic hormone (ACTH), cortisol, and use of statins or antiplatelet agents. All samples were collected from fasting blood. Body mass index (BMI) was computed as body weight in kilograms (kg) divided by height in square meters (m^2^). CAD was diagnosed by coronary CT angiography (CTA) or coronary angiography. Diabetes mellitus was defined in patients who had fasting serum glucose concentration ≥7.0 mmol/L, oral glucose tolerance test results (2-h serum glucose concentration ≥11.1 mmol/L), non-fasting serum glucose concentration ≥11.1 mmol/L, or use of anti-diabetic medication.

### Laboratory Measurements

PAC was measured by radioimmunoassay using a commercially available kit (Beckman Coulter, Brea, CA, USA), and the intra- and inter-assay coefficients of variation were 5.6% and 8.5%, respectively. PRA was measured with an iodine angiotensin I radioimmunoassay kit (Northern Biotechnology Institutes, Beijing, China), and the intra- and inter-assay coefficients of variation were below 10% and 15%, respectively. Blood samples were collected mid-morning after patients had been ambulant for at least 2 h and seated for 15 min. The details of the measurements are described in previous studies from our center (34). Other biochemical indicators were measured by enzymatic methods using an autoanalyzer (7600-010 Automatic Analyzer: Hitachi Medical Systems, Suzhou, China).

### Assessment of White Matter Lesions

**Figure 2:**
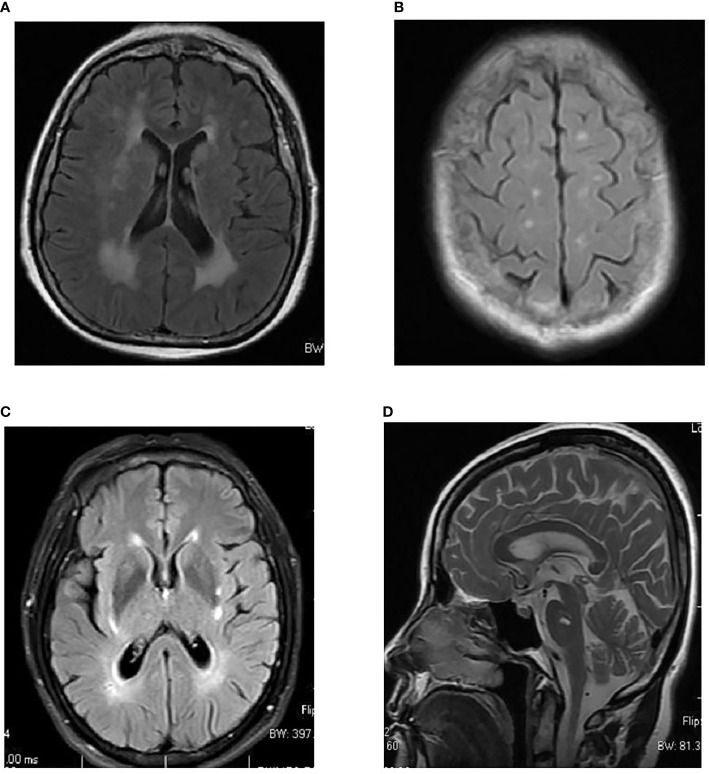
Examples of topographical distribution of white matter lesions in MRI. **(A)** Periventricular white matter lesions (WMLs) (pvWMLs). **(B)** Subcortical WMLs (sWMLs). **(C)** Basal ganglia WMLs (bgWMLs). **(D)** Infratentorial WMLs (iWMLs).

Image data were retrospectively collected from our hospital’s picture archiving and communication system (PACS). T2-FLAIR or FLAIR images for white matter lesion assessments were acquired using a scanner from GE and with magnetic field intensity (1.5 T, n = 154 person-time). WMLs were diagnosed independently by two experienced radiologists. After collection of the images of patients’ WMLs, a modified Scheltens’ scale (0–84 scores) was used to manually score the MRI images of the patients’ WMLs by two experienced radiologists (if the two scores were inconsistent, we recorded the average of the two scores). The periventricular WMLs (pvWMLs), subcortical WMLs (sWMLs), basal ganglia WMLs (bgWMLs), and infratentorial WMLs (iWMLs) were scored separately using the modified Scheltens’ scale. The periventricular high signals (0–6 scores) were scored as follows: the hat-shaped high intensity in the occipital lobe and frontal lobe were both scored as 0–2, and the banded high signals in the lateral ventricle were scored as 0–2. No lesion, lesions ≤5 mm, and lesions with a size of 6–10 mm were scored as 0, 1, and 2, respectively. Subcortical white matter was scored 0–24 as follows: WMLs in the frontal, parietal, occipital, and temporal lobes were rated 0–6. No abnormality was scored 0; the lesions smaller than or equal to 3 mm and the number of less than or equal to 5 were scored 1; the lesions smaller than or equal to 3 mm and the number of more than 6 were scored 2; the lesions within 4–10 mm and the number of less than or equal to 5 were scored 3; the lesion within 4–10 mm and the number equal to or more than 6 were scored 4; the lesions larger than 11 mm and the number of more than 1 were scored 5; and the fused lesions were scored 6. The basal ganglia evaluate the caudate nucleus, lenticular nucleus, globus pallidus, thalamus, and internal capsule; and the infratentorial area evaluates the cerebellum, midbrain, pons, and medulla oblongata. The scoring standards for each part of the above two areas are the same as those for periventricular white matter (35) (**Figure 2**).

### Statistical Analysis

We used R 4.1.1 and SPSS 25.0 to analyze the data. Categorical variables are expressed as frequencies and proportions, and the chi-square test was used to assess significant differences. Normally distributed data were tested by the Kolmogorov–Smirnov D, described by the mean ± SD and compared with Student’s t-test. Non-normally distributed variables are presented as the median ± interquartile range (IQR) and were compared with the Mann–Whitney U test. We analyzed PAC, PRA, and ALD–renin ratio (ARR) continuous and categorical variables (tertiles). In multivariable logistic regression analysis, we analyzed the relationship between PAC, PRA, ARR, and WMLs after adjusting for potential confounding factors (age, sex, BMI, smoking or alcohol consumption status, history of CAD, Cr, HbA1c, TG, TC, LDL-C, HDL-C, Hcy, ACTH, cortisol, DBP, and use of statins and antiplatelet agents). In addition to this model, we further adjusted the history of diabetes mellitus, SBP, and duration of hypertension. In linear regression analysis, PRA, PAC, and ARR were log-transformed. After adjustment for confounding factors, linear regression analysis was used to analyze the correlation between the modified Scheltens’ scale score of WMLs and log(PRA), log(PAC), and log(ARR). We used R with the “mediation” package to estimate the total, direct, and indirect effects of PAC, SBP/DBP, and white matter lesion load. The analyses were corrected for all possible confounding factors. To evaluate the potential effect modification, stratified analyses were further assessed according to age (30–50 or 51–64 years), sex, BMI (<28 or ≥28 kg/m^2^), estimated glomerular filtration rate (eGFR) (<90 or ≥90 ml/min/1.73 m^2^), and PRA (<1 or and ≥1 ng/Ml/h). *p*-Value <0.05 was considered statistically significant.

## Results

### Baseline Characteristics of Study Subjects

At the analysis stage, we included 1,094 participants with ages ranging from 30 to 64 years, of which 547 patients had WMLs and 547 patients had no WMLs as the control group. The baseline characteristics of the patients included in the study are shown in **Table 1** according to WMLs and the control group. No significant differences were noted between the two groups, such as age, sex, BMI, smoking status, alcohol consumption, history of CAD, TG, HbA1c, FBG, ACTH, cortisol, and use of statins. However, patients with WMLs have higher prevalence of diabetes mellitus; lower levels of TC, LDL-C, and PRA; higher levels of Cr, PAC, ARR, Hcy, SBP, and DBP; longer duration of hypertension; and more use of antiplatelet agents than the control group.

**Table 1:** The clinical characteristic of WMLs and no-WMLs group.

	All (n=1094)	WMLs (n=547)	No-WMLs (n=547)	OR,95%CI	*p*. ratio	*P* Value
Age	57.0 [51.0,61.0]	57.0 [51.0,61.0]	57.0 [51.0,61.0]	1.00 [0.98,1.02]	1.000	1.000
Sex						1.000
Male (n, %)	678 (62.0%)	339 (62.0%)	339 (62.0%)	Ref.	Ref.	
Female (n, %)	416 (38.0%)	208 (38.0%)	208 (38.0%)	1.00 [0.78,1.28]	1.000	
BMI (Kg/m^2^)	27.0 [24.6,29.4]	27.1 [24.6,29.5]	26.9 [24.6,29.1]	1.01 [0.98,1.04]	0.496	0.290
Smoking status (n, %)						0.479
Never	734 (67.1%)	361 (66.0%)	373 (68.2%)	Ref.	Ref.	
Current/ Former	360 (32.9%)	186 (34.0%)	174 (31.8%)	1.10 [0.86,1.42]	0.441	
Alcohol consumption (n, %)						0.841
Never	778 (71.1%)	387 (70.7%)	391 (71.5%)	Ref.	Ref.	
Former/current	316 (28.9%)	160 (29.3%)	156 (28.5%)	1.04 [0.80,1.35]	0.790	
Diabetes mellitus (n, %)	274 (25.0%)	152 (27.8%)	122 (22.3%)	1.34 [1.02,1.77]	**0.037**	**0.043**
CAD (n, %)	196 (17.9%)	104 (19.0%)	92 (16.8%)	1.16 [0.85,1.58]	0.346	0.386
Cr (umol/l)	67.8 [57.4,79.4]	69.0 [58.8,82.7]	65.9 [55.7,77.4]	1.01 [1.01,1.02]	**<0.001**	**<0.001**
TG (mmol/L)	1.6 [1.1,2.2]	1.6 [1.1,2.1]	1.6 [1.2,2.3]	0.88 [0.79,0.98]	**0.016**	0.071
TC (mmol/L)	4.5 [3.8,5.1]	4.4 [3.7,5.0]	4.5 [4.0,5.1]	0.86 [0.76,0.97]	**0.016**	**0.016**
LDL-C (mmol/L)	2.7 [2.1,3.2]	2.6 [2.1,3.2]	2.8 [2.2,3.3]	0.84 [0.73,0.97]	**0.015**	**0.018**
HDL-C (mmol/L)	1.0 [0.9,1.2]	1.0 [0.9,1.2]	1.0 [0.9,1.2]	1.73 [1.09,2.75]	0.019	**0.042**
HbA1c (%)	5.9 [5.6,6.3]	5.9 [5.6,6.3]	5.9 [5.6,6.2]	1.06 [0.96,1.17]	0.239	0.542
FBG (mmol/L)	5.0 [4.5,5.6]	5.0 [4.5,5.5]	4.9 [4.5,5.7]	1.02[0.97,1.08]	0.440	0.637
PAC (ng/dl)	13.9 [12.0,20.4]	14.5 [12.1,22.0]	13.6 [11.9,18.2]	1.03 [1.01,1.05]	**<0.001**	**0.002**
Q1(<12.48)	364 (33.3%)	167 (30.5%)	197 (36.0%)	Ref.	Ref.	**0.007**
Q2(12.48-17.26)	365 (33.4%)	173 (31.6%)	192 (35.1%)	1.06 [0.79,1.42]	0.682	
Q3(>17.26)	365 (33.4%)	207 (37.8%)	158 (28.9%)	1.54 [1.15,2.07]	**0.003**	
PRA( ng/ml*h)	1.5 [0.5,2.6]	1.2 [0.4,2.4]	1.8 [0.8,2.8]	0.88 [0.82,0.94]	**<0.001**	**<0.001**
Q1(>2.19)	365 (33.4%)	162 (29.6%)	203 (37.1%)	Ref.	Ref.	**<0.001**
Q2(0.80-2.19)	363 (33.2%)	155 (28.3%)	208 (38.0%)	0.93 [0.70,1.25]	0.647	
Q3(<0.80)	366 (33.5%)	230 (42.0%)	136 (24.9%)	2.12 [1.58,2.85]	**<0.001**	
ARR (ng/dl per ng/ml*h)	10.2 [5.9,27.4]	12.6 [6.3,45.2]	8.7 [5.6,17.9]	1.01 [1.01,1.02]	**<0.001**	**<0.001**
Q1(<6.96)	363 (33.2%)	151 (27.6%)	212 (38.8%)	Ref.	Ref.	**<0.001**
Q2(6.96-18.59)	363 (33.2%)	161 (29.4%)	202 (36.9%)	1.12 [0.83,1.50]	0.455	
Q3(>18.59)	368 (33.6%)	235 (43.0%)	133 (24.3%)	2.48 [1.84,3.34]	**<0.001**	
Serum potassium (mmol/l)	3.8 [3.6,4.1]	3.7 [3.5,4.0]	3.9 [3.7,4.1]	0.31 [0.23,0.43]	**<0.001**	**<0.001**
Hcy (umol/l)	13.4 [10.9,16.8]	14.3 [11.4,17.7]	12.7 [10.5,15.8]	1.05 [1.03,1.07]	**<0.001**	**<0.001**
ACTH (pg/ml)	32.7 [22.1,39.2]	32.7 [22.6,39.5]	32.5 [21.6,39.0]	1.00 [0.99,1.01]	0.978	0.668
Cortisol (ug/dl)	13.5 [8.7,15.9]	13.5 [9.7,16.2]	13.5 [8.0,15.4]	1.02 [1.00,1.04]	**0.045**	0.051
Duration of hypertension (years)	5.0 [1.0,10.0]	6.0 [2.0,12.0]	3.0 [1.0,10.0]	1.05 [1.03,1.06]	**<0.001**	**<0.001**
SBP (mmHg)	147.0 [132.0,163.0]	150.0 [136.0,170.0]	144.0 [130.0,158.0]	1.01 [1.01,1.02]	**<0.001**	**<0.001**
DBP (mmHg)	90.0 [80.0,100.0]	90.0 [80.0,101.0]	88.0 [80.0,97.0]	1.02 [1.01,1.02]	**<0.001**	**<0.001**
Use of medications						
Statins (n, %)	75 (6.9%)	43 (7.9%)	32 (5.9%)	1.37 [0.85,2.22]	0.191	0.232
Antiplatelet agents (n, %)	130 (11.9%)	82 (15.0%)	48 (8.8%)	1.83 [1.26,2.69]	**0.001**	**0.002**

BMI, body mass index; CAD, coronary artery disease; Cr, creatinine; HbA1c, hemoglobin A1c; FBG, fasting blood glucose; PRA, plasma renin activity; PAC, plasma aldosterone concentration; ARR, aldosterone–renin ratio; TG, triglyceride; TC, total cholesterol; LDL-C, low-density lipoprotein-cholesterol; HDL-C, high-density lipoprotein-cholesterol; Hcy, homocysteine; ACTH, adrenocorticotrophic hormone; SBP, systolic blood pressure; DBP, diastolic blood pressure; WMLs, white matter lesions.The bold represents P value < 0.05.

### Logistic Regression Analysis

In logistic regression analysis, we dichotomized the PAC, PRA, and ARR as to whether subjects were in the third tertile (Q3) versus second tertile (Q2) and first tertile (Q1). In a crude model, patients who were in Q3 (>17.26 ng/dl) of PAC (OR 1.54, 95% CI 1.15, 2.07), Q3 (<0.80 ng/dl) of PRA (OR 2.12, 95% CI 1.58, 2.85), and Q3 (>18.59 ng/dl per ng/ml*h) of ARR (OR 2.48, 95% CI 1.84, 3.34) had a significantly higher risk of WMLs than those in Q1 (<12.48) of PAC, Q1 (>2.19) of PRA, and Q1 (<6.96) of ARR. After adjustment for age, sex, BMI, smoking or alcohol consumption status, history of CAD or diabetes mellitus, Cr, TC, TG, LDL-C, HDL-C, HbA1c, Hcy, ACTH, cortisol, SBP, DBP, duration of hypertension, and use of statins or antiplatelet agents, patients who were in Q3 of PAC (OR 1.59, 95% CI 1.15, 2.19), Q3 of PRA (OR 2.50, 95% CI 1.81, 3.44), and Q3 of ARR (OR 2.90, 95% CI 2.10, 4.01) had a significantly higher risk of WMLs than those in Q1 of PAC, Q1 of PRA, and Q1 of ARR (**Table 2**).

**Table 2:** The relationship of PAC, PRA, and ARR with the risk WMLs in logistic regression analysis.

Variables	Model 1		*p-*Value	Model 2		*p*-Value	Model 3		*p*-Value
	OR	95% CI		OR	95% CI		OR	95% CI	
PAC (ng/dl)									
Q1 (<12.48)	Ref.			Ref.			Ref.		
Q2 (12.48–17.26)	1.06	0.79, 1.42	0.682	1.19	0.87, 1.61	0.277	1.16	0.84, 1.58	0.368
Q3 (>17.26)	1.54	1.15, 2.07	**0.003**	1.69	1.24, 2.32	**0.001**	1.59	1.15, 2.19	**0.005**
PRA (ng/ml*h)									
Q1 (>2.19)	Ref.			Ref.			Ref.		
Q2 (0.80–2.19)	0.93	0.70, 1.25	0.647	1.00	0.74, 1.37	0.978	1.09	0.79, 1.50	0.611
Q3 (<0.80)	2.12	1.58, 2.85	**<0.001**	2.32	1.69, 3.18	**<0.001**	2.50	1.81, 3.44	**<0.001**
ARR (ng/dl per ng/ml*h)									
Q1 (<6.96)	Ref.			Ref.			Ref.		
Q2 (6.96–18.59)	1.12	0.83, 1.50	0.455	1.15	0.84, 1.57	0.390	1.17	0.85, 1.61	0.335
Q3 (>18.59)	2.48	1.84, 3.34	**<0.001**	2.84	2.07, 3.91	**<0.001**	2.90	2.10, 4.01	**<0.001**

Model 1: unadjusted. Model 2: adjusted for age, sex, BMI, smoking or alcohol consumption status, history of coronary artery disease, Cr, TC, TG, LDL-C, HDL-C, HbA1c, Hcy, ACTH, cortisol, diastolic blood pressure, and use of statins and antiplatelet agents. Model 3: Model 2 + adjusted for systolic blood pressure, duration of hypertension, and history of diabetes mellitus.

PAC, plasma aldosterone concentration; PRA, plasma renin activity; ARR, aldosterone–renin ratio; WMLs, white matter lesions; Cr, creatinine; TC, total cholesterol; TG, triglyceride; LDL-C, low-density lipoprotein-cholesterol; HDL-C, high-density lipoprotein-cholesterol; HbA1c, hemoglobin A1c; Hcy, homocysteine; ACTH, adrenocorticotrophic hormone.

The bold represents P value < 0.05.

### Linear Regression Analysis

**Figure 3:**
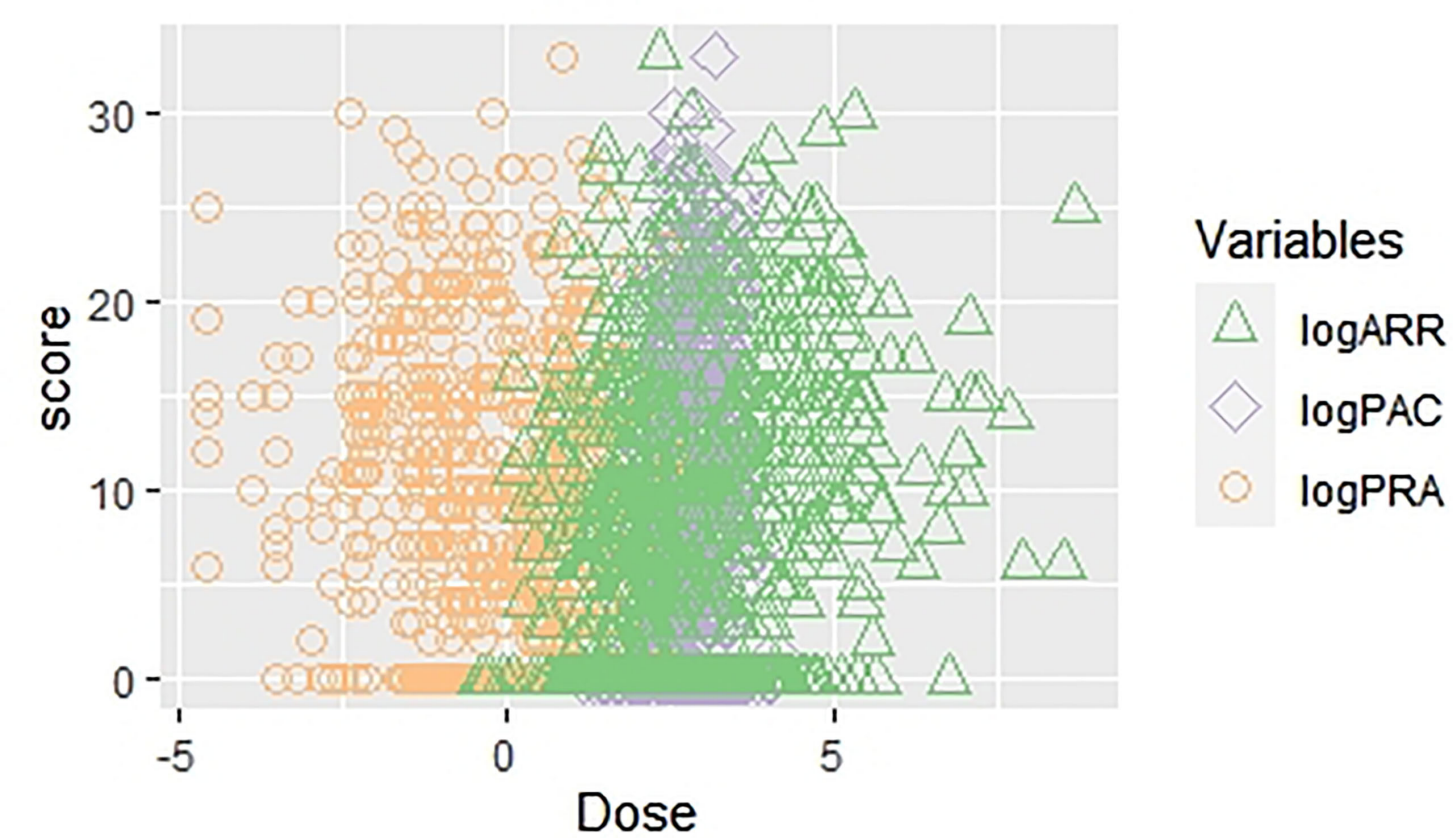
The scatter plot regarding univariate linear regression [WMLs score *vs*. log(PAC), log(PRA), and log(ARR)]. WMLs, white matter lesions; PAC, plasma aldosterone concentration; PRA, plasma renin activity; ARR, aldosterone–renin ratio.

We analyzed PAC, PRA, and ARR continuously and evaluated the correlation between the modified Scheltens’ scale score and the variables by using the linear regression analysis. We separately analyzed the correlation between the score and related parameters, considering their multicollinearity. In a crude model, log(PAC) (β = 2.75; 95% CI 1.67, 3.84; *p* < 0.001), log(PRA) (β = −1.60; 95% CI −1.95, −1.25; *p* < 0.001), and log(ARR) (β = 1.78; 95% CI 1.44, 2.12; *p* < 0.001) were all significantly correlated with white matter lesion load. After adjustment of all possible confounding factors, log(PAC) (β = 2.36; 95% CI 1.30, 3.41; *p* < 0.001), log(PRA) (β = −1.76; 95% CI −2.09, −1.43; *p* < 0.001), and log(ARR) (β = 1.86; 95% CI 1.55, 2.17; *p* < 0.001) were still correlated with white matter lesion load (**Table 3** and **Figure 3**). In the multiple linear regression analysis, we also examined the topographical distribution of white matter lesion load in with PRA, PAC, and ARR after adjusting for confounders. In addition to the lack of a relationship between log(PAC) and iWMLs, log(PAC) was positively associated with pvWMLs and sWMLs. Log(ARR) was also positively associated with pvWMLs, sWMLs, bgWMLs, and iWMLs. In contrast, log(PRA) was negatively associated with pvWMLs, sWMLs, bgWMLs, and iWMLs (**Table 4**).

**Table 3:** Correlation between PAC, PRA, ARR, and score of WMLs in linear regression analyses.

Variables	Model 1				Model 2				Model 3			
	Coefficient	SE	*p*-Value	95% CI	Coefficient	SE	*p*-Value	95% CI	Coefficient	SE	*p*-Value	95% CI
Log(PAC) ng/dl	2.75	0.552	**<0.001**	1.67, 3.84	2.71	0.543	**<0.001**	1.64, 3.77	2.36	0.535	**<0.001**	1.30, 3.41
Log(PRA) ng/ml*h	−1.60	0.180	**<0.001**	−1.95, −1.25	−1.72	0.173	**<0.001**	−2.06, −1.39	−1.76	0.168	**<0.001**	−2.09, −1.43
Log(ARR) ng/dl per ng/ml*h	1.78	0.172	**<0.001**	1.44, 2.12	1.86	0.164	**<0.001**	1.54, 2.18	1.86	0.160	**<0.001**	1.55, 2.17

Model 1: unadjusted. Model 2: adjusted for age, sex, BMI, smoking or alcohol consumption status, history of coronary artery disease, Cr, TC, TG, LDL-C, HDL-C, HbA1c, Hcy, ACTH, cortisol, diastolic blood pressure, and use of statins or antiplatelet agents. Model 3: Model 2 + adjusted for systolic blood pressure, duration of hypertension, and history of diabetes mellitus.

PAC, plasma aldosterone concentration; PRA, plasma renin activity; ARR, aldosterone–renin ratio; WMLs, white matter lesions; BMI, body mass index; Cr, creatinine; TC, total cholesterol; TG, triglyceride; LDL-C, low-density lipoprotein-cholesterol; HDL-C, high-density lipoprotein-cholesterol; HbA1c, hemoglobin A1c; Hcy, homocysteine; ACTH, adrenocorticotrophic hormone.

The bold represents P value < 0.05.

**Table 4:** Correlation between PAC, PRA, ARR, and score of pvWMLs, sWMLs, bgWMLs, and iWMLs in linear regression analyses.

Variables	pvWMLs*				sWMLs*			
	Coefficient	SE	*p*-Value	95% CI	Coefficient	SE	*p*-Value	95% CI
Log(PAC) ng/dl	0.78	0.215	**<0.001**	0.42, 1.14	1.11	0.236	**0.002**	0.65, 1.58
Log(PRA) ng/ml*h	−0.60	0.058	**<0.001**	−0.72, −0.49	−0.66	0.075	**0.001**	−0.81, −0.51
Log(ARR) ng/dl per ng/ml*h	0.64	0.055	**<0.001**	0.53, 0.74	0.72	0.072	**<0.001**	0.58, 0.86
**Variables**	bgWMLs*				iWMLs *			
	**Coefficient**	**SE**	***p*-Value**	**95% CI**	**Coefficient**	**SE**	***p*-Value**	**95% CI**
Log(PAC) ng/dl	0.32	0.127	**0.012**	0.07, 0.57	0.15	0.096	0.132	−0.04, 0.33
Log(PRA) ng/ml*h	−0.31	0.040	**<0.001**	−0.39, −0.23	−0.19	0.031	**<0.001**	−0.25, −0.13
Log(ARR) ng/dl per ng/ml*h	0.32	0.039	**<0.001**	0.24, 0.39	0.19	0.030	**<0.001**	0.13, 0.25

WMLs, white matter lesions; pvWMLs, periventricular WMLs; sWMLs, subcortical WMLs; bgWMLs, basal ganglia WMLs; iWMLs, infratentorial WMLs; BMI, body mass index; Cr, creatinine; TC, total cholesterol; TG, triglycerides; LDL-C, low-density lipoprotein-cholesterol; HDL-C, high-density lipoprotein-cholesterol; HbA1c, hemoglobin A1c; Hcy, homocysteine; ACTH, adrenocorticotrophic hormone.

^*^Adjusted for age, sex, BMI, Cr, smoking or alcohol consumption status, history of coronary artery disease or diabetes mellitus, TC, TG, LDL-C, HDL-C, HbA1c, Hcy, ACTH, cortisol, diastolic blood pressure, systolic blood pressure, duration of hypertension, and use of statins or antiplatelet agents.

The bold represents P value < 0.05.

### Mediation Analysis

**Figure 4:**
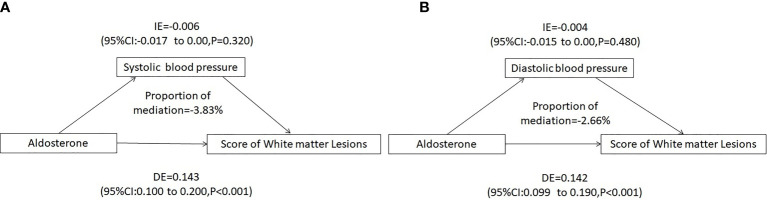
Mediating analysis of systolic or diastolic blood pressure on PAC and white matter lesion Load. **(A)** Systolic blood pressure mediated a -3.83% association between PAC and white matter lesion load, **(B)** Diastolic blood pressure mediated a -2.66% association between PAC and white matter lesion load. Adjusted for age, sex, BMI, Cr, smoking or alcohol consumption status, history of coronary artery disease or diabetes mellitus, TC, TG, LDL-C, HDL-C, HbA1c, Hcy, ACTH, cortisol, and use of statins or antiplatelet agents. IE, indirect effect; DE, direct effect; BMI, body mass index; Cr, creatinine; TC, total cholesterol; TG, triglycerides; LDL-C, low-density lipoprotein-cholesterol; HDL-C, high-density lipoprotein-cholesterol; HbA1c, hemoglobin A1c; Hcy, homocysteine; ACTH, adrenocorticotrophic hormone.

Since ALD would predict the total WMLs score, simple mediation analyses were conducted to detect potential mediating effects of hypertension. We estimated that SBP or DBP mediated −3.83% or −2.66% of the association between increasing PAC and white matter lesion load, respectively (**Figure 4**).

### Stratified Analyses

**Figure 5:**
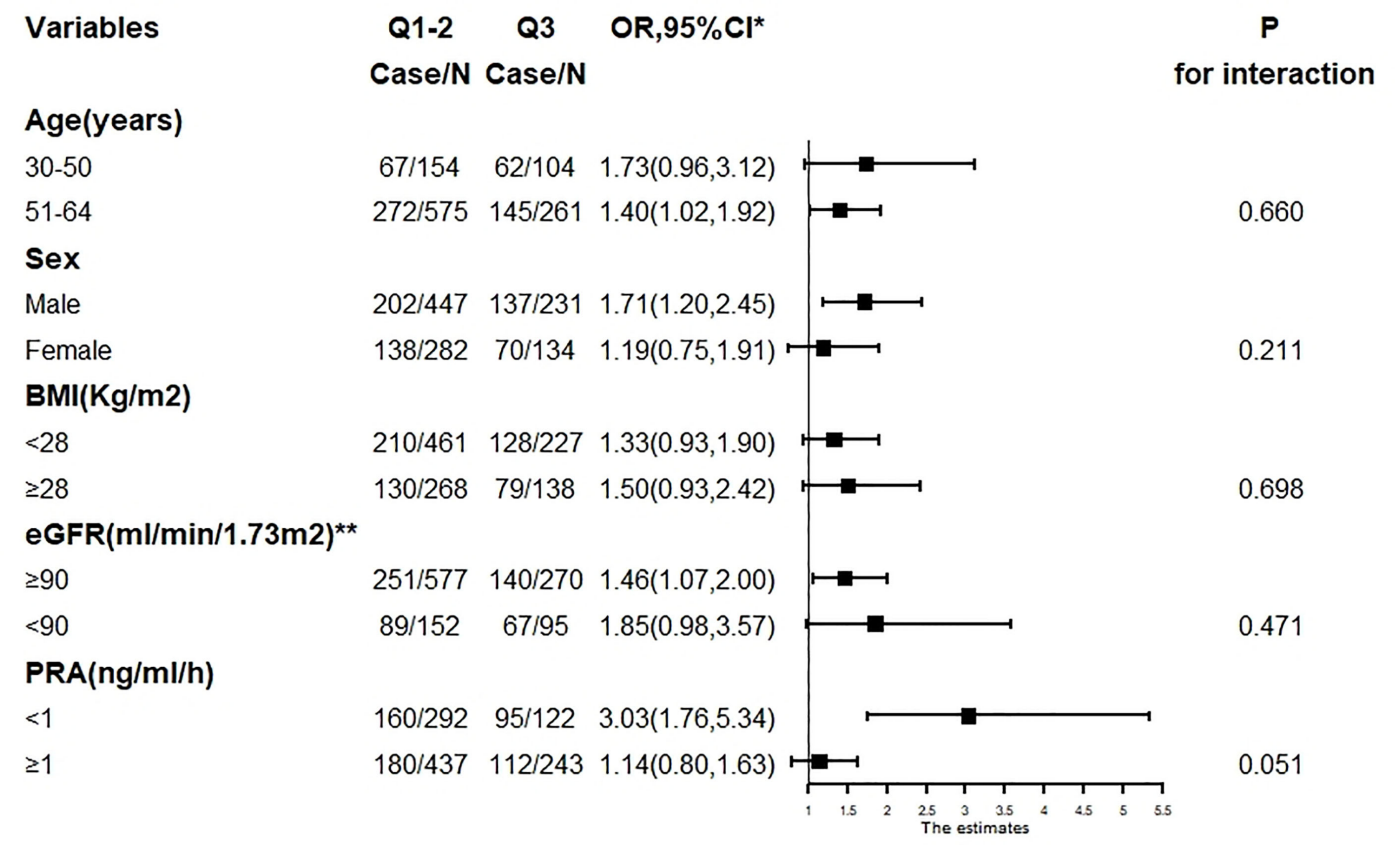
The association between PAC (Q3 compared with Q1–2) and WMLs in various subgroups. PAC, plasma aldosterone concentration; WMLs, white matter lesions; BMI, body mass index; Cr, creatinine; TC, total cholesterol; TG, triglyceride; LDL-C, low-density lipoprotein-cholesterol; HDL-C, high-density lipoprotein-cholesterol; HbA1c, hemoglobin A1c; Hcy, homocysteine; ACTH, adrenocorticotrophic hormone. ^*^Adjusted, if not stratified, for age, sex, BMI, Cr, smoking or alcohol consumption status, history of coronary artery disease or diabetes mellitus, TC, TG, LDL-C, HDL-C, HbA1c, Hcy, ACTH, cortisol, diastolic blood pressure, systolic blood pressure, duration of hypertension, and use of statins or antiplatelet agents. ^**^Chronic Kidney Disease Epidemiology Collaboration (CKD-EPI) equation.

There was no evidence of subgroup heterogeneity regarding the change in risk of WMLs (*p* > 0.05 for interaction for all). None of the other variables, including age, sex, BMI, eGFR, and PRA, showed a significant effect modification on the PAC–WMLs association (**Figure 5**).

## Discussion

In our study, the higher PAC and ARR and lower PRA increased the risk of WMLs. Furthermore, PAC and ARR were positively associated with white matter lesion load, and PRA was negatively associated with white matter lesion load. We also reached the same conclusion when we studied the topographical distribution of WMLs in relation to PRA, PAC, and ARR. These relationships between PRA, ARR, and WMLs were associated with higher PAC.

Several studies have found that higher PAC increases stroke (36), coronary artery calcium (37), heart failure (38), acute myocardial infarction (39), and renal impairment (40). Moreover, the association between ALD and atherosclerosis has been widely shown in humans, while no research has focused on the relationship between ALD and WMLs. This is the first study to focus on the association between ALD and WMLs in humans.

Dinh et al. found that ALD increases oxidative stress in the cerebral artery, and mRNA expression of the proinflammatory cytokine chemokine ligands CCL7 and CCL8 and interleukin (IL)-1β in the brain. These increases are prevented by spironolactone treatment in endothelial cell MR-deficient mice (32). *CYP11B2* gene, which determines ALD synthesis, has been found in vascular endothelial cells, smooth muscle cells, cardiomyocytes, liver, kidney, brain, and other tissues. Verpillat et al. observed an association between *CYP11B2* gene (especially the T allele) and WMLs (33). Similarly, MR is expressed extensively in the CNS (41, 42). The function of the MR in the CNS is to mediate diverse behavioral responses, including memory and affect (43).

An animal model study confirmed that spironolactone could prevent vascular remodeling and anti-fibrosis (44). Pires et al. found that MR antagonism can reduce cerebral artery remodeling and white matter injury in those fed high fat diet without lowering blood pressure (31). Many studies have proven that hypertension is the main risk factor for WMLs (45, 46). In particular, lower DBP levels are related to lower white matter lesion load (47), but the pathological mechanism is not clear.

Our findings showed that PAC was not associated with bgWMLs or iWMLs but positively associated with pvWMLs and sWMLs. First, WMLs occur frequently in pvWMLs and sWMLs but rarely in iWMLs and bgWMLs. Second, it may be related to MR expression being low in infratentorial and basal ganglia areas and high in periventricular and subcortical areas. However, further animal models are needed to prove this result.

The mediation analysis showed that ALD contributes to white matter lesion load through SBP or DBP, but the mediation effect was low. Notably, most of the effects on WMLs could be explained independently of diastolic or SBP. Our results also highlighted the importance of considering the potential burden of elevated ALD levels on the cerebral small vessel not through hypertension.

Meanwhile, our stratified analyses showed no significant effect modification on the ALD–WML association, especially because a high level of ALD causes WMLs regardless of PRA, which could emphasize the effect of ALD and MR on WMLs. Hyperaldosteronism can occur either as primary aldosteronism (renin-independent) or secondary aldosteronism (renin-dependent).As the most common cause of secondary hypertension, primary aldosteronism is associated with increased cardiovascular risk. It is well known that ALD increases the risk of acute cardiovascular events both directly and indirectly (mostly mediated by hypertension) (43–45). Moreover, the association between ALD and atherosclerosis has also been widely shown in humans and animal models (48–50). However, no research has focused on the relationship between ALD and WMLs. We showed that ALD contributed to the white matter lesion load independent of SBP or DBP, which can help understand the effects of ALD on the nervous system, and can also increase awareness of primary aldosteronism screening in non-hypertension departments. In the future, we will evaluate whether spironolactone can decrease white matter lesion load.

There were some limitations in our study: first, the prevalence of WMLs in our hypertension center was less than that in general population because not all patients underwent cerebral MRI scans, which weakened the association of ALD and WMLs. Second, we collected the diagnosis of WMLs from our hospital’s PACS. Only images of patients diagnosed with WMLs were obtained by two radiologists, and we collected the images of patients with WMLs and were manually scored by two experienced radiologists using the modified Scheltens’ scale. However, some radiologists ignoring mild WMLs failed to indicate the diagnosis of WMLs on MRI, which would underestimate the correlation between ALD and WMLs. Third, we cannot completely rule out possible confounding factors from unmeasured factors. However, we had adjusted for potential confounders, including the most important risk factor for DBP, SBP, and duration of hypertension. In particular, our research subjects were younger than 65 years, so we can better rule out the risk factors for age.

## Conclusion

A higher level of PAC increased the risk of WMLs. The PAC was positively associated with white matter lesion load independent of diastolic or SBP. Future studies will focus on the spatial distributions of WMLs on MRI of patients with primary aldosteronism and the effect of spironolactone on WMLs.

## Data Availability Statement

The raw data supporting the conclusions of this article will be made available by the authors, without undue reservation.

## Ethics Statement

The study was approved by the Ethics Committee of the People’s Hospital of Xinjiang Uygur Autonomous Region.

## Author Contributions

YY and NL conceived and designed the present study, carried out the statistical analysis, prepared the original manuscript, and revised the submitted version. NL participated in the acquisition of fund support for the present study. YL, QZ, MH, WZ, XY, DZ, QL, MW, GC, MC, KZ, LW, JH, and NM participated in controlling the quality of the cases. All authors contributed to the article and approved the submitted version.

## Funding

The study was financially supported by Non-profit Central Research Institute Fund of Chinese Academy of Medical Sciences (2019PT330003).

## Conflict of Interest

The authors declare that the research was conducted in the absence of any commercial or financial relationships that could be construed as a potential conflict of interest.

## Publisher’s Note

All claims expressed in this article are solely those of the authors and do not necessarily represent those of their affiliated organizations, or those of the publisher, the editors and the reviewers. Any product that may be evaluated in this article, or claim that may be made by its manufacturer, is not guaranteed or endorsed by the publisher.

## References

[1] Wardlaw JM Smith EE Biessels GJ Cordonnier C Fazekas F Frayne R. Neuroimaging Standards for Research Into Small Vessel Disease and ITS Contribution to Ageing and Neurodegeneration. Lancet Neurol (2013) 12(8):822–38. doi: 10.1016/S1474-4422(13)70124-8 PMC3714437 23867200

[2] Caligiuri ME Perrotta P Augimeri A Rocca F Quattrone A Cherubini A. Automatic Detection of White Matter Hyperintensities in Healthy Aging and Pathology Using Magnetic Resollauce Knagmg: A Review. Neuromformatics (2015) 13(31):261–76. doi: 10.1007/s1202l-015-9260-v PMC4468799 25649877

[3] Pantoni L. Cerebral Small Vessel Disease: From Pathogenesis and Clinical Characteristics to Therapeutic Challenges. Lancet Neurol (2010) 9(7):689–701. doi: 10.1016/S1474-4422(10)70104-6 20610345

[4] Gouw AA Seewann A van der Flier WM Barkhof F Rozemuller AM Scheltens P. Heterogeneity of Small Vessel Disease: A Systematic Review of MRI and Histopathology Correlations. J Neurol Neurosurg Psychiatry (2011) 82(2):126–35. doi: 10.1136/jnnp.2009.204685 20935330

[5] Kloppenborg RP Nederkoorn PJ Geerlings MI van den Berg E. Presence and Progression of White Matter Hyperintensities and Cognition: A Meta-Analysis. Neurology (2014) 82:2127–38. doi: 10.1212/WNL.0000000000000505 24814849

[6] Hainsworth AH Minett T Andoh J Forster G Bhide I Barrick TR. Neuropathology of White Matter Lesions, Blood-Brain Barrier Dysfunction, and Dementia. Stroke (2017) 48(10):2799–804. doi: 10.1161/STROKEAHA.117.018101 PMC5986073 28855392

[7] Coutu JP Goldblatt A Rosas HD Salat DH. Alzheimer’s Disease Neuroimaging Initiative (ADNI) White Matter Changes Are Associated With Ventricular Expansion in Aging, Mild Cognitive Impairment, and Alzheimer’s Disease. J Alzheimers Dis (2016) 49(2):329–42. doi: 10.3233/JAD-150306 PMC5996384 26444767

[8] Wang L Leonards CO Sterzer P Ebinger M. White Matter Lesions and Depression: A Systematic Review and Meta-Analysis. J Psychiatr Res (2014) 56:56–64. doi: 10.1016/j.jpsychires.2014.05.005 24948437

[9] Kuller LH Longstreth WT Jr Arnold AM Bernick C Bryan RN Beauchamp NJ Jr. Cardiovascular Health Study Collaborative Research Group. White Matter Hyperintensity on Cranial Magnetic Resonance Imaging: A Predictor of Stroke. Stroke (2004) 35:1821–5. doi: 10.1161/01.STR.0000132193.35955.69 15178824

[10] Wolfson L Wakefield DB Moscufo N Kaplan RF Hall CB Schmidt JA. Rapid Buildup of Brain White Matter Hyperintensities Over 4 Years Linked Toambulatory Blood Pressure, Mobility, Cognition, and Depression in Old Persons. J Gerontol A Biol Sci Med Sci (2013) 68(11):1387–94. doi: 10.1093/gerona/glt072 PMC3805298 23766429

[11] McNeil CJ Myint PK Sandu AL Potter JF Staff R Whalley LJ. Blood Pressure and White-Matter Disease Progression in a Biethnic Cohort: Atherosclerosis Risk in Communities (ARIC) Study. Stroke (2010) 41:3–8. doi: 10.1161/STROKEAHA.109.566992 19926835 PMC2803313

[12] McNeil CJ Myint PK Sandu AL Potter JF Staff R Whalley LJ. Increased Diastolic Blood Pressure Is Associated With MRI Biomarkers of Dementia-Related Brain Pathology in Normative Ageing. Age Ageing (2018) 47:95–100. doi: 10.1093/ageing/afx102 29106439

[13] Aribisala BS Morris Z Eadie E Thomas A Gow A Valdés Hernández MC. Blood Pressure, Internal Carotid Artery Flow Parameters, and Age-Related White Matter Hyperintensities. Hypertension (2014) 63:1011–8. doi: 10.1161/HYPERTENSIONAHA.113.02735 PMC3984108 24470459

[14] Shokouhi M Qiu D Samman Tahhan A Quyyumi AA Hajjar I. Differential Associations of Diastolic and Systolic Pressures With Cerebral Measures in Older Individuals With Mild Cognitive Impairment. Am J Hypertens (2018) 31:1268–77. doi: 10.1093/ajh/hpy104 PMC6233687 30052724

[15] Gottesman RF Schneider AL Albert M Alonso A Bandeen-Roche K Coker L. Midlife Hypertention and 20-Year Cognitive Change: The Atherosclerosis Risk in Communities Neurocognitive Study. JAMA Neurol (2014) 21287:1–10. doi: 10.1001/jamaneurol.2014.1646 PMC4226067 25090106

[16] Meschia JF Bushnell C Boden-Albala B Braun LT Bravata DM Chaturvedi S. Guidelines for the Primary Prevention of Stroke: A Statement for Healthcare Professionals From the American Heart Association/American Stroke Association. Stroke (2014) 45:3754–832. doi: 10.1161/STR.0000000000000046 PMC5020564 25355838

[17] Walker KA Sharrett AR Wu A Schneider ALC Albert M Lutsey PL. Association of Midlife to Late-Life Blood Pressure Patterns With Incident Dementia. JAMA (2019) 322:535–45. doi: 10.1001/jama.2019.10575 PMC6692677 31408138

[18] Sierra C. Hypertension and the Risk of Dementia. Front Cardiovasc Med (2020) 7:5. doi: 10.3389/fcvm.2020.00005 32083095 PMC7005583

[19] Artham S Fouda AY El-Remessy AB Fagan SC. Vascular Protective Effects of Angiotensin Receptor Blockers: Beyond Blood Pressure. Receptors Clin Investig (2015) 2(3):e774. doi: 10.14800/rci.774 PMC4548973 26317114

[20] Fouda AY Artham S El-Remessy AB Fagan SC. Renin-Angiotensin System as a Potential Therapeutic Target in Stroke and Retinopathy: Experimental and Clinical Evidence. Clin Sci (Lond) (2016) 130(4):221–38. doi: 10.1042/CS20150350 26769658

[21] Royea J Hamel E. Brain Angiotensin II and Angiotensin IV Receptors as Potential Alzheimer’s Disease Therapeutic Targets. Geroscience (2020) 42(5):1237–56. doi: 10.1007/s11357-020-00231-y PMC7525853 32700176

[22] Jackson L Eldahshan W Fagan SC. Within the Brain: The Renin Angiotensin System. Ergul A Int J Mol Sci (2018) 19(3):876. doi: 10.3390/ijms19030876 PMC5877737 29543776

[23] Haspula D Clark MA. Molecular Basis of the Brain Renin Angiotensin System in Cardiovascular and Neurologic Disorders: Uncovering a Key Role for the Astroglial Angiotensin Type 1 Receptor AT1R. J Pharmacol Exp Ther (2018) 366(2):251–64. doi: 10.1124/jpet.118.248831 29752427

[24] Hoffmann N Peters J. Functions of the (Pro) Renin Receptor (Atp6ap2) at Molecular and System Levels: Pathological Implications in Hypertension, Renal and Brain Development, Inflammation, and Fibrosis. Pharmacol Res (2021) 173:105922. doi: 10.1016/j.phrs.2021.105922 34607004

[25] Mulatero P Verhovez A Morello F Veglio F. Diagnosis and Treatment of Low-Renin Hypertension. Clin Endocrinol (Oxf) (2007) 67(3):324–34. doi: 10.1111/j.1365-2265.2007.02898.x 17573898

[26] Athimulam S Lazik N Bancos I. Low-Renin Hypertension. Endocrinol Metab Clin North Am (2019) 48(4):701–15. doi: 10.1016/j.ecl.2019.08.003 31655771

[27] Funder JW. Primary Aldosteronism: Present and Future. Vitam Horm (2019) 109:285–302. doi: 10.1016/bs.vh.2018.10.006 30678860

[28] Funder JW Carey RM Mantero F Murad MH Reincke M Shibata H. The Management of Primary Aldosteronism: Case Detection, Diagnosis, and Treatment: An Endocrine Society Clinical Practice Guideline. J Clin Endocrinol Metab (2016) 101(5):1889–916. doi: 10.1210/jc.2015-4061 26934393

[29] Fuller PJ Young MJ. Mechanisms of Mineralocorticoid Action. Hypertension (2005) 46(6):1227–35. doi: 10.1161/01.HYP.0000193502.77417.17 16286565

[30] Northcott CA Fink GD Garver H Haywood JR Laimon-Thomson EL McClain JL. The Development of Hypertension and Hyperaldosteronism in a Rodent Model of Life-Long Obesity. Endocrinology (2012) 153(4):1764–73. doi: 10.1210/en.2011-1176 PMC3320259 22355066

[31] Pires PW McClain JL Hayoz SF Dorrance AM. Mineralocorticoid Receptor Antagonism Prevents Obesity-Induced Cerebral Artery Remodeling and Reduces White Matter Injury in Rats. Microcirculation (2018) 25(5):e12460. doi: 10.1111/micc.12460 29758591 PMC6117832

[32] Dinh QN Young MJ Evans MA Drummond GR Sobey CG Chrissobolis S. Aldosterone-Induced Oxidative Stress and Inflammation in the Brain Are Mediated by the Endothelial Cell Mineralocorticoid Receptor. Brain Res (2016) 1637:146–53. doi: 10.1016/j.brainres.2016.02.034 26923165

[33] Verpillat P Alpérovitch A Cambien F Besançon V Desal H Tzourio C. Aldosterone Synthase (CYP11B2) Gene Polymorphism and Cerebral White Matter Hyperintensities. Neurology (2001) 56(5):673–5. doi: 10.1212/wnl.56.5.673 11245725

[34] Luo Q Li NF Yao XG Zhang DL Abulikemu SF Chang GJ. Potential Effects of Age on Screening for Primary Aldosteronism. J Hum Hypertens (2016) 30(1):53–61. doi: 10.1038/jhh.2015.21 25880592

[35] Scheltens P Barkhof F Leys D Pruvo JP Nauta JJ Vermersch P. A Semiquantative Rating Scale for the Assessment of Signal Hyperintensities on Magnetic Resonance Imaging. J Neurol Sci (1993) 114(1):7–12. doi: 10.1016/0022-510x(93)90041-v 8433101

[36] Ohno Y Sone M Inagaki N Yamasaki T Ogawa O Takeda Y. Prevalence of Cardiovascular Disease and Its Risk Factors in Primary Aldosteronism: A Multicenter Study in Japan. Hypertension (2018) 71(3):530–7. doi: 10.1161/HYPERTENSIONAHA.117.10263 29358460

[37] Inoue K Goldwater D Allison M Seeman T Kestenbaum BR Watson KE. Serum Aldosterone Concentration, Blood Pressure, and Coronary Artery Calcium: The Multi-Ethnic Study of Atherosclerosis. Hypertension (2020) 76(1):113–20. doi: 10.1161/HYPERTENSIONAHA.120.15006 PMC10681368 32418495

[38] Güder G Bauersachs J Frantz S Weismann D Allolio B Ertl G. Complementary and Incremental Mortality Risk Prediction by Cortisol and Aldosterone in Chronic Heart Failure. Circulation (2007) 115:1754–61. doi: 10.1161/CIRCULATIONAHA.106.653964 17372171

[39] Beygui F Collet JP Benoliel JJ Vignolles N Dumaine R Barthélémy O. High Plasma Aldosterone Levels on Admission Are Associated With Death in Patients Presenting With Acute ST-Elevation Myocardial Infarction. Circulation (2006) 114:2604–10. doi: 10.1161/CIRCULATIONAHA.106.634626 17116769

[40] Kawashima A Sone M Inagaki N Takeda Y Itoh H Kurihara I. Renal Impairment Is Closely Associated With Plasma Aldosterone Concentration in Patients With Primary Aldosteronism. Eur J Endocrinol (2019) 181(3):339–50. doi: 10.1530/EJE-19-0047 31319380

[41] Fuller PJ Verity K. Mineralocorticoid Receptor Gene Expression in the Gastrointestinal Tract: Distribution and Ontogeny. J Steroid Biochem (1990) 36(4):263–7. doi: 10.1016/0022-4731(90)90215-e 2168006

[42] Fuller PJ Yang J Young MJ. Mechanisms of Mineralocorticoid Receptor Signaling. Vitam Horm (2019) 109:37–68. doi: 10.1016/bs.vh.2018.09.004 30678864

[43] Joe¨ls M de Kloet ER. 30 YEARS OF THE MINERALOCORTICOID RECEPTOR: The Brain Mineralocorticoid Receptor: A Saga in Three Episodes. J Endocrinol (2017) 234(1):T49–66. doi: 10.1530/JOE-16-0660 28634266

[44] Baldo MP Zaniqueli D Forechi L Machado RC Rodrigues SL Mill JG. Effects of Spironolactone in Spontaneously Hypertensive Adult Rats Subjected to High Salt Intake. Clinics (Sao Paulo) (2011) 66(3):477–82. doi: 10.1590/s1807-59322011000300020 PMC3072011 21552676

[45] Dufouil C deKersaint-Gilly A Besançon V Levy C Auffray E Brunnereau L. Longitudinal Study of Blood Pressure and White Matter Hyperintensities: The EVA MRI Cohort. Neurology (2001) 56(7):921–6. doi: 10.1212/wnl.56.7.921 11294930

[46] Godin O Tzourio C Maillard P Mazoyer B Dufouil C. Antihypertensive Treatment and Change in Blood Pressure Are Associated With the Progression of White Matter Lesion Volumes: The Three-City (3c)-Dijon Magnetic Resonance Imaging Study. Circulation (2011) 123(3):266–73. doi: 10.1161/CIRCULATIONAHA.110.961052 21220733

[47] Caunca MR Simonetto M Cheung YK Alperin N Lee SH Elkind MSV. Diastolic Blood Pressure Is Associated With Regional White Matter Lesion Load: The Northern Manhattan Study. Stroke (2020) 51(2):372–8. doi: 10.1161/STROKEAHA.119.025139 PMC7219602 31910743

[48] Monticone S D’Ascenzo F Moretti C Williams TA Veglio F Gaita F. Cardiovascular Events and Target Organ Damage in Primary Aldosteronism Compared With Essential Hypertension: A Systematic Review and Meta-Analysis. Lancet Diabetes Endocrinol (2018) 6:41–50. doi: 10.1016/S2213-8587(17)30319-4 29129575

[49] Keidar S Kaplan M Pavlotzky E Coleman R Hayek T Hamoud S. Aldosterone Administration to Mice Stimulates Macrophage NADPH Oxidase and Increases Atherosclerosis Development. Circulation (2004) 109:2213–20. doi: 10.1161/01.CIR.0000127949.05756.9D 15123520

[50] McGraw AP Bagley J Chen WS Galayda C Nickerson H Armani A. Aldosterone Increases Early Atherosclerosis and Promotes Plaque Inflammation Through a Placental Growth Factor-Dependent Mechanism. J Am Heart Assoc (2013) 2:e000018. doi: 10.1161/JAHA.112.000018 23525413 PMC3603255

