# Supplementary material for: Positive Association Between Plasma Aldosterone Concentration and White Matter Lesions in Patients With Hypertension
Source: Front Endocrinol (Lausanne). 2021 Nov 18;12:753074. doi: 10.3389/fendo.2021.753074 (PMC8637536; doi:10.3389/fendo.2021.753074)
Supplement: Supplementary file 1 [file Table_1.docx]

**Table S1 The clinical characteristic of WMLs and no-WMLs group**

|  | All（n=10033） | WMLs (n=547) | No-WMLs (n=9486) | *Z/χ^2^* Value | *P* Value |
| --- | --- | --- | --- | --- | --- |
| Age | 48(42,54) | 57(51,61) | 47(42,53) | -20.511 | **<0.001** |
| Sex |  |  |  |  |  |
| Male (n, %) | 6240,62.19 | 339,61.97 | 5901,62.21 |  |  |
| Female (n, %) | 3793,31.81 | 208,38.03 | 3585,37.79 | 0.012 | 0.913 |
| BMI (Kg/m^2^) | 26.88(24.49,29.39) | 26.93(24.46,29.54) | 26.88(24.49,29.39) | -0.157 | 0.875 |
| Smoking status (n, %) |  |  |  |  |  |
| Never | 6433,64.12 | 361,66.00 | 6072,64.01 | - | **-** |
| Current/ Former | 3600,35.88 | 186,34.00 | 3414,35.99 | 0.887 | 0.346 |
| Alcohol consumption (n, %) |  |  |  |  |  |
| Never | 6519,64.98 | 387,70.75 | 6132,64.64 |  |  |
| Former/current | 3514,35.02 | 160,29.25 | 3354,35.36 | 8.475 | **0.004** |
| Diabetes mellitus (n, %) | 1654,16.49 | 152,27.79 | 1502,15.83 | 53.679 | **<0.001** |
| CAD (n, %) | 1075,10.71 | 104,19.01 | 971,10.24 | 41.643 | **<0.001** |
| Cr (umol/l) | 66.75(56.6,77.30) | 69.00(58.80,83.23) | 66.60(56.50,77.00) | -4.822 | **<0.001** |
| TG (mmol/L) | 1.58(1.1.14,2.29) | 1.55(1.10,2.11) | 1.58(1.14,2.30) | -2.324 | **0.020** |
| TC (mmol/L) | 4.49(3.90,5.11) | 4.37(3.74,5.00) | 4.50(3.91,5.11) | -2.864 | **0.004** |
| LDL-C (mmol/L) | 2.71(2.18,3.24) | 2.63(2.06,3.17) | 2.72(2.18,3.25) | -2.544 | **0.011** |
| HDL-C (mmol/L) | 1.01(0.86,1.19) | 1.00(0.87,1.19) | 1.01(0.86,1.19) | -0.604 | 0.546 |
| Hb1c (%) | 5.70(5.40,6.10) | 5.80(5.50,6.40) | 5.70(5.40,6.10) | -6.138 | **<0.001** |
| FBG (mmol/L) | 4.79(4.38,5.37) | 4.94(4.47,5.70) | 4.78(4.37,5.35) | -4.738 | **<0.001** |
| PAC (ng/dl) | 14.65(12.23,20.94) | 17.55(13.15,24.47) | 13.80(11.94,19.12) | -22.670 | **<0.001** |
| Q1(<12.80) | 3358,33.47 | 192,35.11 | 3166,33.38 |  |  |
| Q2(12.80-18.42) | 3327,33.16 | 163,29.80 | 3164,33.35 |  |  |
| Q3(>18.42) | 3348,33.37 | 192,35.10 | 3156,33.27 | 2.951 | 0.229 |
| PRA (ng/Ml*h) | 1.45(0.42,2.65) | 0.30(0.12,0.64) | 2.13(1.15,3.12) | -2.615 | **0.009** |
| Q1(<0.66) | 3362,33.51 | 207,37.84 | 3155,33.26 |  |  |
| Q2(0.66-2.22) | 3322,33.11 | 184,33.64 | 3138,33.08 |  |  |
| Q3(>2.22) | 3349,33.38 | 156,28.52 | 3193,33.66 | 7.385 | **0.025** |
| ARR (ng/Dl per ng/Ml*h) | 10.83(5.92,34.24) | 60.35(26.04,133.78) | 7.63(5.10,13.14) | -61.783 | **<0.001** |
| Q1(<7.16) | 3339,33.28 | 155,28.34 | 3184,33.57 |  |  |
| Q2(7.16-22.50) | 3339,33.28 | 181,33.09 | 3158,33.29 |  |  |
| Q3(>22.50) | 3350,33.39 | 211,38.57 | 3139,33.09 | 8.908 | **0.012** |
| Serum potassium (mmol/l) | 3.79(3.54,4.03) | 3.74(3.46,4.00) | 3.80(3.55,4.03) | -3.717 | **<0.001** |
| Hcy (umol/l) | 12.45(10.28,15.90) | 14.06(11.30,17.78) | 12.40(10.23,15.76) | -7.481 | **<0.001** |
| ACTH (pg/ml) | 33.60(22.80,40.35) | 32.70(22.50,39.50) | 33.60(22.80,40.40) | -0.931 | 0.352 |
| Cortisol (ug/dl) | 13.47(8.08,16.27) | 13.47(9.65,16.22) | 13.47(7.98,16.27) | -1.900 | 0.057 |
| Duration of hypertension(years) | 3.00(0.90,7.00) | 6.00(2.00,12.00) | 3.00(0.80,7.00) | -12.076 | **<0.001** |
| SBP(mmHg) | 144(130,158) | 150(136,170) | 143(130,158) | -7.632 | **<0.001** |
| DBP(mmHg) | 90(80,100) | 90(80,101) | 90(80,100) | -2.055 | **0.040** |
| Use of medications |  |  |  |  |  |
| Statins (n, %) | 491,4.89 | 43,7.86 | 448,4.72 | 10.944 | **0.001** |
| Antiplatelet agents(n, %) | 737,7.35 | 82,14.99 | 655,6.90 | 49.682 | **<0.001** |

[BMI: Body Mass Index](http://shortof.com/suolueci/bmi-body-mass-index), CAD: Coronary Artery Disease, Cr: creatinine, HbA1C: Hemoglobin A1C, FBG: Fast Blood Glucose; PRA: Plasma renin activity, PAC: Plasma aldosterone concentration, ARR: Aldosterone-renin ratio, TG: triglycerides, TC: total cholesterol, LDL-C: Low density lipoprotein-cholesterol, HDL-C: High density lipoprotein-cholesterol, Hcy: Homocysteine, ACTH: adrenocorticotrophic hormone, SBP: Systolic blood pressure, DBP: Diastolic blood pressure.
